# Transnational Lessons from Mexican-Origin Border Crossing Future Teachers: Decolonizing Teacher Practices

**DOI:** 10.3390/bs15101413

**Published:** 2025-10-17

**Authors:** Irasema Mora-Pablo, G. Sue Kasun, Zurisaray Espinosa, J. Nozipho Moyo

**Affiliations:** 1 Department of Languages, University of Guanajuato, Guanajuato 36000, Mexico; 2 Department of Middle & Secondary Education, Georgia State University, Atlanta, GA 30302, USA; skasun@gsu.edu (G.S.K.); jmoyo2@gsu.edu (J.N.M.); 3 Department of Inclusive Education, Kennesaw State University, Kennesaw, GA 30144, USA; zespinos@kennesaw.edu

**Keywords:** decolonization, transnationals, narratives, linguistic funds of knowledge, teacher preparation programs

## Abstract

Grounded in frameworks of decoloniality and transnationalism, this study examines how organizational behaviors in education—particularly in teacher preparation—can shift to more inclusively serve transnational youth, challenging Eurocentric, nation-bound assumptions about pedagogy, belonging, and professional development. The present study aims to understand Mexican-origin returnees and transnational migrants who came back to Mexico to pursue English teacher preparation degrees in Guanajuato and Hidalgo after spending significant periods of time on either side of the Mexico-U.S. border. Our study aimed to recognize and describe the experiences that shaped their English teaching practices and professional commitments to teaching English as a foreign language. Using narrative inquiry within a longitudinal qualitative study of 28 Mexican-origin pre-service English teachers, our research was guided by frameworks of decoloniality and transnationalism. Our findings reveal that for participants, U.S.-based teaching approaches were recalled most often as the best compared to Mexican ones. Participants also reflected on how their experiences of learning and adapting to a new culture contributed to their professional identity and how their ability to adapt constituted a form of international-mindedness. We argue that through the comparison and adoption of multiple decolonial practices, teacher preparation programs can produce culturally responsive pedagogies that cross borders. By illustrating how teacher preparation programs can cultivate culturally responsive pedagogies that transcend national boundaries, the study highlights the potential of decolonial and transnational perspectives to transform organizational behavior at multiple levels of educational practice and policy.

## 1. Introduction

Migration between the United States and Mexico has long had deep economic, political, social, and cultural implications, particularly in shaping educational landscapes on both sides of the border. This article examines the experiences of Mexican-origin returnees and transnational migrants who have lived on both sides of the border for a number of years and returned to Mexico to pursue English teaching degrees in Guanajuato and Hidalgo. While substantial research has focused on transnational youth in U.S. schools (Bartlett, 2014; García, 2011; Suárez-Orozco, 2001), less attention has been paid to those who return to Mexico to become educators. This article aims to fill that gap in order to provide insights about how multilingual teachers draw from unique resources based on lived experiences, as well as draw connections to broader border-crossing work all educators are called to do (Gándara & Jensen, 2021; Kleyn & Porter, 2021; Mora-Pablo et al., 2015; Zúñiga & Saucedo, 2019).

In this article, a Mexican-based and U.S.-based researchers focus on 28 pre-service English teachers in two Mexican public universities who reflected on issues such as their origin and cultural environment, and teaching experiences as transnationals. These participants and their families resided for a number of years in the United States, always maintaining close ties to their country of origin, Mexico, until they decided to or were forced to return. These individuals spent parts of their lives in both countries and returned to Mexico with advanced English language skills, which positions them advantageously within Mexico’s current K-12 English teaching workforce (Mora-Pablo & Basurto, 2019).

The work by Toohey and Smythe (2022) emphasizes that decolonial teacher education demands a fundamental reconsideration of both educator preparation methods as well as their intended objectives. This standpoint encourages us to examine prevailing epistemologies and Western-centered methodologies while questioning deficit narratives assigned to diverse students who speak different languages and come from various cultures. The proposed teacher education model should focus on social justice principles while embracing epistemic diversity and acknowledging the actual experiences of transnational groups. The importance of this decolonial transition becomes clear when we examine transnational students and families who gather complex and situated forms of knowledge through migration which remain frequently unrecognized. González et al. (2005) defined these elements as ‘funds of knowledge’. This educational framework pushes teachers to value the deep experiential understanding migrant students possess which comes from their experiences across different languages and cultures as well as institutional settings. Decoloniality in teacher education involves more than just incorporating diverse materials into current teaching programs. We propose a deep structural transformation that utilizes students’ existing knowledge while training educators to become co-constructors of knowledge with their students instead of mere transmitters of fixed content. The main objective of this study was to identify and interpret those events that have had an impact on their teaching practice, and also to understand how these events (discriminatory at times on both sides of the border) helped them become teachers in the first place.

## 2. Migration and Transnationalism in Mexico and the U. S.

To contextualize the complexities of transnational students’ educational experiences, it is important to understand how migration and transnationalism have been studied on both sides of the border. Using decolonization as a lens, we can re-signify teaching practices to better support the adjustment of transnationals into the Mexican educational system. In this section, we review literature on migration and transnationalism, then move on to a discussion of decolonizing language education and connect these ideas to funds of knowledge and the role they play in learning for transnational students.

In the U.S., research on migration and transnationalism has highlighted both the challenges and adaptations of Mexican immigrants. For example, in the U. S., some scholars have focused their attention on narrative work and chronicles of border crossing to examine various aspects of self-presentation among first-generation Mexican immigrants (De Fina, 2003, 2009; De Fina et al., 2021). Scholars like Relaño Pastor (2007) have examined how *transfronterizo* (cross-border) students navigate their experiences in cross-border schooling contexts, while Jacobo Suárez et al. (2022) have explored the educational struggles faced by transnational children who have to overcome obstacles when trying to pursue their education. Additionally, Gándara and Jensen (2020) focus on how educators can be better prepared to support transnational mobility in education.

In Mexico, studies have also focused on school adaptation and the challenges of integrating transnational students into local systems (Jacobo Suárez et al., 2022; Hamann & Zúñiga, 2011; Panait & Zúñiga, 2016; Zúñiga & Hamann, 2021). The fundamental goal of these studies has been to look at the impact and importance of language teachers on students, as well as the potential repercussions of this influence, such as long-term beneficial or negative effects (Mora-Pablo & Ocampo-Márquez, 2024; Trejo Guzmán et al., 2016). Although the migration phenomenon poses a challenge for both countries, on an individual level, children who are subjected to constant movement between countries face the daily challenges of adjusting to a new language, culture, and society’s frequent rejection.

Glick Schiller et al. (1992) describe transnationalism as “the processes by which immigrants build social fields that link together their country of origin and their country of settlement” (p. 1). These fields include family, society, economy, religion and politics. Although this definition frames transnationalism as a collective process, other authors have emphasized the individual experiences involved. Therefore, transnationalism is not simply a label, but a term that captures the lived experiences of individuals. These experiences encompass “relationships that span two or more nations, including sustained and meaningful flows of people, money, labor, goods, information, advice, care, and love; in addition, systems of power (i.e., patriarchy, Westernism) can be created or reinforced in this process” (Sánchez, 2007, p. 493). This perspective, at an individual level, highlights the complexities of the phenomenon, with agency as a and lived experiences crucial aspects to consider.

Transnationalism “has reconstructed localities, regrouping, as a result of the mobility of both people and ideas, the practices and meanings derived from multiple geographical and historical points of origin” (Rizvi, 2019, p. 27). More specifically, Hamann et al. (2008) describe transnational students in Mexico as those “who look Mexican in appearance, whose parents may be Mexican, but who may not be fully proficient in Spanish, who may not have been socialized to Mexican expectations of the student/teacher relationship” (p. 62). Research has examined how transnationals use their knowledge to benefit from the system (Cortez Román & Hamann, 2014; Mora-Pablo et al., 2015; Perez, 2012; Zúñiga & Hamann, 2021), while others have highlighted their invisibility and struggles with assimilation or incorporation (Delaunay & Santibañez, 1997; Hamann et al., 2006; Tacelosky, 2013; Zúñiga & Saucedo, 2019). These examples demonstrate the challenges that transnationals face when attempting to connect with one country, language, or culture over another. Transnational students gain experience and information as they go back and forth, forming their linguistic and cultural knowledge funds, which we might refer to as transnational knowledge funds. A transnational student “is one who has transnational experiences of moving to, from, and in between two nation states and educational contexts whilst acquiring knowledges and shaping his or her imagination of self and belongingness in the process” (Serna-Gutiérrez, 2019, p. 20). To gain a deeper understanding of their experiences and knowledge, we now explore the concept of decolonizing teacher education.

## 3. Theoretical Framework: Decolonizing Teacher Education and Experiences as Funds of Knowledge

Scholars of transnationalism frequently engage with decoloniality in their work (e.g., Kasun et al., 2023; Walcott, 2020). This article also considers decoloniality as both a goal and a method, not unlike revered border theorist’s Gloria Anzaldúa’s refrain of, “I change myself, I change the world” (Anzaldúa, 2015, p. 113). Anzaldúa’s work was one of the first points of entry into conceiving both the complex power structures of borderlands and hybrid identities which emerge from lives that span multiple cultures that are situated among various nexuses of power (Anzaldúa, 2015). In teacher education, decolonial approaches involve rethinking how educators are prepared and for what purposes, as well as reconsidering the methods used to prepare them (Toohey & Smythe, 2022), particularly in terms of how they engage their own and their students’ identities. Decoloniality refers to the project of uncoupling from the “colonial matrix of power,” wherein doing and being are not separate and where Western colonial processes are called into question. Furthermore, Tuck and Yang’s (2012) assertion that “decolonization is not a metaphor” highlights the need to liberate not just land but also individuals from limiting borders. This framing challenges discourses such as “native speakerism” which holds “native” English speakers’ forms and accents as the ideal standard, ignoring the global diversity of English (Pennycook, 2002, 2017; Motha, 2014) and how the transnational movements of people create an often decolonizing aspect of deterritorializing some geographies (Christiansen, 2017).

English language education as a global project has been taken on to further coloniality; its use has served as a tool of power and exclusion (e.g., Motha, 2014; Pennycook, 2002, 2017). Kumaravadivelu (2016) speaks to the need to address each context toward the decolonial instead of offering broad-strokes prescriptions for *all* teacher preparation programs. Toohey and Smythe (2022) suggest that ELA teacher education should challenge the colonial logics of “mastery” and be redesigned to reflect the local realities and environments in which students and teachers operate. Their call is to recognize learners’ backgrounds and the ways they learn English as integral to the development of teacher education programs, building on Kumaravadivelu’s (2016) ideas.

### Funds of Knowledge

During their ongoing migration between the two countries, transnationals accumulate a wealth of experiences and knowledge. Scholars have referred to these knowledges as funds of knowledge, which enable them to navigate different cultural and social spaces (González et al., 2005; Hamann et al., 2008; Sánchez, 2007). For this research, transnational bilingualism is characterized by the language variety of students in both Spanish and English, which is frequently perceived as deficient in academic contexts due to the disparities in their language proficiency in both languages (Serna Gutiérrez & Mora-Pablo, 2017; Trejo Guzmán et al., 2016). These perceived deficits, especially in Spanish, are often intensified by teachers’ limited understanding of how to address them effectively (Serna Gutiérrez & Mora-Pablo, 2017). P. H. Smith (2001) frames this concept as *funds of linguistic knowledge*. In the Mexican classroom, these sources of linguistic knowledge are typically invisible, as other scholars have emphasized (e.g., Tacelosky, 2013). This highlights a lack of educational empathy for transnational students, as well as the failure to meet their linguistic needs. Moreover, English classrooms in Mexico tend not to promote new learning opportunities for transnational students, as their English proficiency frequently exceeds that of their peers (Tacelosky, 2013). This further exemplifies the existence of funds of cultural knowledge, which encompass both social and cultural capital. Bourdieu (1991) defines cultural capital as “knowledge, skills, and other cultural acquisitions, as exemplified by educational or technical qualifications” (p. 14), while Coleman (1988) describes social capital as the resources and networks people use to achieve specific goals. Together, these forms of capital play a crucial role in shaping individual opportunities and social mobility.

Lastly, transnational students possess varying degrees of biculturality (Petrón & Greybeck, 2014; Tacelosky, 2018) and bilingualism and biliteracy (Gándara & Jensen, 2021; Hamann et al., 2006; Hornberger, 2007; de la Piedra, 2011). Therefore, it is crucial to develop a conceptual framework for the knowledge sets of transnational students. The Funds of Knowledge approach highlights the collective skills and experiences these students have acquired through their transnational lives, in both the U.S. and Mexico. These funds simultaneously confront deficit-based educational models while offering a comprehensive perspective on student contributions to the classroom environment. Educators who recognize students’ diverse funds will create responsive teaching methods that support identity affirmation while advancing student achievement within multilingual and multicultural educational settings.

## 4. Methodology

This study aimed to answer the following research question: What are the educational system memories that these transnationals have of their teachers on both sides of the border, and what is the significance of these experiences to participants in their self-perception as future English teachers? The study employed a qualitative methodology. This methodology “begins with the assumptions and the use of interpretative/theoretical frameworks that inform the study of research problems addressing the meaning individuals or groups ascribe to a social or human problem” (Creswell, 2013, p. 44). We employed a narrative inquiry approach within the guiding theoretical frameworks of decoloniality and transnationalism in a longitudinal study of 28 Mexican-origin pre-service English teachers. This approach, particularly suited for examining decolonialism and transnationalism, allows participants to express their experiences across borders, languages, and cultural contexts (Bamberg & Georgakopoulou, 2008; De Fina & Georgakopoulou, 2015). Narrative inquiry is a qualitative research approach that focuses on understanding experience through the stories people live and tell. As articulated by Clandinin and Connelly (2022), it is both a methodology and the phenomenon of study, wherein researchers gather, analyze, and retell stories to explore the complexities of a person’s lived experiences within their social, cultural, and institutional contexts.

By utilizing autobiographies (Walker, 2017) and semi-structured interviews, participants and researchers collaboratively made sense of their narratives (Stolz & Ozoliņš, 2018). Autobiographies, as a tool of research, allows for the participant to create a more in-depth set of reflections than what might be generated by interviews alone. The semi-structured interviews, however, provide for focused areas of inquiry alongside thoughtful probing by the interviewer to more deeply understand certain points and themes.

The study began in 2017 and continued through 2024, investigating key events and understandings that shaped participant’s teaching practices. The autobiographies (coded as AB) detailed each participant’s journey to becoming an English teacher, while interviews (coded as INT) conducted in English, Spanish, or both languages further enriched the data collection. These instruments allowed for a more in-depth exploration of data, providing a comprehensive understanding of the participant’s transnational experiences (Clandinin & Connelly, 2022).

## 5. Context and Participants

Guanajuato and Hidalgo are part of a historically significant migratory zone in Mexico. According to Durand (2017), this region shares five key characteristics: (1) it has one of the largest migrant populations; (2) it accounts for more than half of all Mexican migrants; (3) it has the highest number of migrants with legal status in the United States; (4) it receives a substantial percentage of economic remittances; and (5) it is home to extensive social networks, migrant associations, and established migration routes. These features contribute to migratory patterns that are often classified as return migration or transnationalism, wherein individuals move fluidly between Mexico and the United States. Migrants from these states often maintain strong transnational connections, participating in social, economic, and cultural exchanges between places of origin and destinations in the U.S.

The 28 participants in this study were considered transnationals, following Petron’s definition, which describes transnationals as individuals who “essentially have two home bases,” maintaining close ties with family members and communities on both sides of the border (Frausto-Hernández, 2017, p. 2). These participants have established and sustained relationships across borders, particularly in the areas of family, education and community. For the purposes of this publication, we selected a subset of these participants to highlight the specific challenges they face in becoming English teachers in Mexico. Approximately half of the participants were born in Mexico, while the other half were born in the United States. All had spent at least two years, and in many cases much longer, attending U.S. public schools, during which they acquired significant proficiency in English. Upon returning to Mexico, most transitioned to public schools, often experiencing a greater fluency in English than in Spanish. The majority returned for family-related reasons, such as reunification or economic considerations. Some returned due to major family transitions, like the death of a family member, and subsequently were unable to return to the U.S. due to legal or familial complications. The participants ranged in age from 20 to 35 years old, reflecting a diverse set of experiences with migration, bilingualism, and transnational context. All participants signed letters of informed consent, and their names have been changed to protect their identity.

## 6. Data Analysis

Participants were asked to write an autobiography, followed by individual interviews. The autobiographies focused on their journeys to becoming English language teachers, foregrounding any experiences they had across borders. This focus on transnational experiences was an unusual discursive move, as most Mexican-origin pre-service teachers were typically perceived and characterized by their teacher educators as simply “Mexican,” overlooking the complexity of backgrounds and who they are. The research, therefore, created a new space for these teachers to self-author themselves. Then, two interviews were carried out in English, Spanish or bilingually, depending on the participants’ preferences at any given time. For the purposes of this article, we translated extracts that were originally in Spanish. Data analysis followed an on-going process, involving frequently revisiting the autobiographies and the interview transcriptions. Our coding followed an iterative approach, utilizing “constant comparative analysis” which ultimately led us to identify several overarching themes (Saldaña, 2021). We applied paradigmatic cognition, defined as “classifying a particular instance as belonging to a category or concept” (Polkinghorne, 1995, p. 9) by identifying similarities in the data and grouping them into categories.

The authors of this article are transnational. The two lead researchers have collected data in Mexico for decades. One is of Mexican origin, currently based in Mexico, with extensive experience living abroad, and is married to a partner from another country. The other is a bilingual White woman who has also lived abroad for many years and is now based in the United States. The two other authors are both from the Global South. One spent her early childhood in Cuba and maintained connections to the country through visits during her youth, while the other grew up speaking the heritage languages of Zimbabwe and has lived in four different countries. Collectively, the authors strive to center decolonial perspective while remaining cognizant of their various privileges. All four scholars have been actively involved in research efforts within Mexico.

Following, we explore the major themes we found based on the participants’ narratives. We examine how mostly U.S.-based school teaching practices and teachers were remembered as more exemplar than Mexican ones, how the pre-service transnational teachers grew and engaged English in terms of considering teaching English, and how the transnational participants’ ability to adapt was perceived as a strength, especially for teaching.

## 7. Findings

In the following sections, we discuss the emerging themes: (1) Transnational schooling and memories of teachers, (2) English and culture learned in the U.S. and advantages for future teachers, and (3) Transnational student’s successful adaptive practices and positive life outlook in Mexico.

### 7.1. Transnational Schooling and Memories of Teachers

One major theme was the participants’ experiences with teachers in the U.S. and Mexico across school contexts. All of the interviewees related similar experiences with being treated better by U.S. teachers and struggling less with school, versus Mexican teachers and struggling more in the Mexican school system. In separate work, we theorize how and why this might be a somewhat misguided sense of decoloniality among Mexicans, which can be understood within the broader context of 500 years of colonial influence (Kasun & Mora-Pablo, 2021; Mignolo, 2000; Quijano, 2007). Isabella (all names are pseudonyms) talked about her negative educational experiences in secondary school in Mexico and how some of her teachers would not regularly show up to class, leaving a classroom of 35 or more students unattended. She compared them to her experiences with one of her teachers in the U.S. who would take her class on trips to theme parks such as SeaWorld and Busch Gardens to help them learn more English by turning them into educational experiences. Isabella reflected that she is currently striving to be the type of teacher that engages her students due to her transnational schooling experiences.

The participants noted that, in the U.S., their English for Speakers of Other Languages (ESOL) classes were generally part of a well-structured system where teachers provided them with more individualized attention, in contrast to their educational experiences in Mexico. Olivia mentioned, “*I think I like more the American school system, because they help you into getting to level and they put more emphasis in your development, for me, since I was always behind,*” (Olivia, INT) and of her educational experiences in Mexico, she mentioned, “s*o here, it was the complete opposite, the teachers don’t have an idea of what to do with you as a returnee*” (Olivia INT). Even though Olivia struggled with keeping up in school in the U.S. due to having to keep up with the English language, and then also struggling with the Spanish language in Mexico, she was able to survive by seeking help from her peers and by adapting across contexts by being resilient.

In a similar way, Moisés was inspired by his U.S. high school teacher to want to become an English teacher, he said. He said the same teacher had already taught two of his siblings in Oklahoma, “*She taught us how to learn English. She helped us with homework, and because she got to [know] all of us… I think that kinda motivates me to become an English teacher to do the same, help other kids, other people learn English also.”* (Moisés, INT). Some other participants learned to use their negative experiences in Mexico to their advantage and turn them into something positive when becoming English teachers. This is the case of Hugo:

*I miss my country [the U.S.] so much and since the trauma I have been through, to teach other children the same that I have been, because it was the most horrible, an extreme thing that I could ever imagine. So, I want to help others that are passing the same thing, as well because I found that the language teacher has many opportunities for a job, like a translator or tourist guide. *

(Hugo, AB)

Hugo’s professional image was deeply rooted in his lived experiences as a language learner, but he wants to go beyond being an English teacher. He saw this profession as a step on his professional career as he has found other possibilities that he would perhaps also like to explore.

The teaching practices and treatment the interviewees experienced from the U.S. teachers were evident in their reflections about their future teachers’ selves and they would hope to adopt with their students. For instance, Lucia mentioned that her education in the U.S. influenced her to be kinder, inspired by a teacher who had shown her kindness. She mentioned that her teacher would tell her “‘*Wake up with a purpose, follow your dreams, don’t give up, and keep on going,’ she always said that with her students. It really encouraged me.*” (Lucía, AB).

At the same time, many participants reflected on their challenges learning English in the U.S. and the support their teachers provided. In contrast, they described greater difficulties with Spanish in Mexico, where they felt teachers lacked understanding of the unique needs of returnee students. Yolanda explained a painful memory, one that mirrors many participants’ stories, “*My aunt was my teacher, and she was going to send me all the way back to kindergarten just for not knowing Spanish!*” (Yolanda, INT). This echoed others’ senses of feeling bullied by several of their within-Mexico teachers.

### 7.2. English and Culture Learned in the U.S. and Advantages for Future Teachers

Participants’ diverse experiences related to language and culture learned in the U.S. broadened their perspectives, allowing them to navigate the challenges they encountered as children, adolescents, and later as adults in a teacher preparation program in Mexico.

While some participants acknowledged moments where they felt self-conscious speaking English in public in Mexico, they also emphasized viewing English as a valuable skill they acquired in the U.S., one that they strived to master for personal and professional success. They expressed a desire to present themselves as approachable and modest, not as *presumidos* (conceited). As Isabella noted, “*You sometimes hear comments that you’re showing off or talking about someone*,” (Isabella, INT), but this did not deter her or others from recognizing the importance of bilingualism.

Furthermore, Julia mentioned that teaching professionally, she would treat returnees well, “*I would send them the message that I identify with them as returnee or as native speakers*” (Julia, AB), because she understands the struggles of returning back to a country in which she feels there is not much hope of a future. She mentioned that she would treat them fairly and not have them teach the class as some teachers did with her, due to her knowing the English language when she went back to Mexico. Elena also reflected on the type of teacher would like to be as a result of her transnational experiences in the U.S. and then tutoring English in Mexico. She said she knew that she could help her students facing particularly difficult circumstances because of her knowledge of the world by pushing them to learn the English language and achieve a better quality of life.

All of the participants in this study viewed their English skills as an advantage within Mexico, because they began to reflect on the opportunities and experiences having knowledge of the English language has afforded them already. For example, Alejandro began to think of becoming an English teacher due to his experiences teaching chess and English to some students which lead him on the path to becoming an English teacher. Such a path opened his mind to continue to think about future possibilities for his life. He expressed:

*I mean, you can do many things, and friends now, that are here in the university and other universities, they tell me, ‘hey, you know, I wish I had that level of English’, so you really, like you have an advantage of that, because as we know, many people here in Mexico, to finish their career, some like engineers, they ask for a certain score in English.*

(Alejandro, INT).

Alejandro had high life aspirations, because he knew what his knowledge of the English language entailed for his future. Other interviewees, like Hugo, mentioned they also used their English to help their family members strive by tutoring them as well. Hugo’s transnational thinking and knowledge of the English language was also an advantage to his family, because his nephew was, at the time, considering learning more English through him, going to the U.S., and earning money for the family. Even though becoming an English teacher was not the first choice for some of the participants, they still used the advantage of knowing English to acquire a stable quality of life through teaching in Mexico, because for some of them, it might have been what they perceived as their only advantage. For example, Elena felt that she became a teacher because English was the only thing she has ever felt like she was good at. She mentioned, “ *I couldn’t study engineering ‘cuz when people ask in Spanish, it’s very hard for me to understand. In certain concepts, like I even, in a meeting at school, they are like talking about the ‘Consejo Técnico’ [Teacher professional development day] and I’m like ‘what’s going on?*” (Elena, INT). In a sense, English became a saving device for some of the participants. In Ana’s case, she felt comfortable with being an English teacher, because she was always able to have work, “*I don’t fight for a job or, to a certain extent, well, we don’t have much competition, which is the good thing and I feel lucky because, well, not everyone has this degree*” (Ana, INT). Ana felt that she was always able to have a stable job, because not everyone is fortunate enough to know English in Mexico. Others, like Julia, saw the economic stability of being an English teacher, but also enjoyed the idea of being a teacher, because of her personal struggles and experiences. Julia mentioned, “*I think I’m trying to help my students have a wider range of what American society has and try to see both sides of the coin, the positive and negative side of it*” (Julia, AB). She felt that she was able to teach students the realities of life and what they needed to do in order to do well in life in Mexico and in the U.S.

### 7.3. Transnational Student’s Successful Adaptive Practices and Positive Life Outlook in Mexico

All of the participants reflected on how they had to continually adapt to new food, cultural practices, contexts, schools, teachers, economic statuses and the English and Spanish language across contexts. Alejandro expressed the following about adapting to life in Mexico: “*For me, adapting was actually trying the food, and for example, getting along with people that are around you, so socializing is very effective when adapting… even though I didn’t know Spanish.”* (Alejandro, INT). Alejandro, as well as Valentina mentioned they had to force themselves to be part of the culture in order to feel comfortable around people by imitating what they were saying and doing. Mateo also had to adapt to the way people spoke and behaved across spaces in the U.S., “*so what I used to do, just like stand behind people when they were like having conversations and just listening to them, and then I started like slowly talking.*” (Mateo, INT). He also had to learn how to reply to text messages in Spanish in order to be able to socialize better with his friends. Nicolás had to adapt to his new economic situation, “y*ou have to limit yourself on the material things of course. You had to adapt yourself to your economic status,*” (Nicolás, INT) especially going back to Mexico, however, he was resilient and creative in his new life by knowing how to adapt to his present situation and by using his English language skills.

Similarly, all of the participants portrayed themselves as independent thinkers. Nicolás reflected, “*I always had that attitude that I want to do things on my own, like not depend on other people, so I had to study to know, to learn, not to be that person, or that guy that has to get help all the time at school.*” (Nicolás, INT). The transnational participants in this study developed a mentality of independence in order to survive across contexts. Some of them learned how to be independent by living in the U.S. and spending more time alone while their parents worked. Like Nicolás, most reflected about living a humbler life in Mexico compared to how they lived in the U.S. On adapting to U.S. culture, Lourdes mentioned that it was important for her to learn English for her to adapt and be able to live in American culture without depending on someone to translate. For others like Javier, learning English was not as enjoyable and felt forced to learn more in order to maintain his job.

Overall, most interviewees felt relieved that they were connecting with their Mexican roots upon return to Mexico. It was difficult, however, in that some of them had to sacrifice their old ways of living in the U.S. to learn new ways of being in Mexico. They generally felt it was a fair trade-off because they were happy being close to family; however, they were not always received well by teachers, extended family members, and by strangers within the community.

The participants’ global experiences lead them to have a global mindset which allowed them to survive in various cultural spaces. The interviewees had a different outlook on life after living in the U.S. and then settling in Mexico due to factors outside of their control. Interviewees reflected they use their international knowledge as cultural and social capital. Alejandro reflected that they [transnationals] “*should feel international because they know the customs and they know how places react to different stimuli or two different things, or their culture and traditions*” (Alejandro, AB). Mateo talked about taking opportunities in his life and not waiting until later to take on new opportunities that might lead him to greater success and stability. Others concentrated on more specific goals. Valentina expressed, similarly representing an openness to a life lived across borders:

*When I finish my [studies], maybe I’m going to have a good job. Okay, I almost think that, but I’m like, If I go to the USA, maybe [I can become] a teacher over there, maybe I’m going to have more money instead of right here in Mexico.*

(Valentina, INT).

Like Valentina, others expressed they would like to go back to the U.S. for more economic advancement, however, they felt like they were realistic about their prospects of being successful in the long term, because they are aware of the hard work and maturity it takes to be successful,

*I know what it’s like, I guess, to live in the States and then here makes me more international, like, I guess yes, because I have a concept or an idea of what it’s like to go and work, and is not as people see it here in Mexico, how they think that you can, if you go to the States, automatically you start to earn money and become a rich person.*

(Mateo, INT).

Some of the participants reflected that having an international mindset brought them more maturity, stability and even better treatment within Mexico. That internationalism came to them, however, after complex adaptation processes in Mexico.

## 8. Discussion

### 8.1. Transnationalism, Decolonizing Teacher Practices, and Their Professional Implications

These participants’ experiences provide crucial insights into how transnational experiences shape both personal and professional growth. Through their migration across the U.S. -Mexico border, they developed unique funds of knowledge (González et al., 2005; Moll et al., 1992) rooted in their bilingual, bicultural, and transnational experiences. These experiences, formed by navigating two distinct educational and cultural systems, positioned them as vital resources in the movement to decolonize education, particularly in English language teaching. Primarily, the pre-service teachers were poised to offer greater understanding and wanted to help build resilience in their students–even uniquely to learning English to build resilience. This deeper understanding and empathy help undo the coloniality of the many ways teaching was experienced in both countries, and especially Mexico, as a kind of difficulty brought on by the erasure of their transnational backgrounds and their weaker Spanish language skills when in Mexico, particularly.

As educators and researchers, it is important to understand that the participants’ reflections on their cross-border experiences informed their teaching practice in significant ways. They carry the weight of historical colonialism, particularly in how English has been used as a tool for subjugation (Pennycook, 2002, 2017). By engaging with their own marginalization, these future teachers are uniquely positioned to decolonize classroom practices and foster more inclusive learning environments. They move beyond simple reflective practice, as Chapman (2011) suggests, and critically engage with how their actions and those of their students interact in ways that can either perpetuate or dismantle systems of oppression.

This process of decolonizing education begins with self-reflection, which demands a significant emotional investment and the willingness to confront personal biases (Díaz-Lázaro, 2011). For these participants, their transnational experiences offer an opportunity to decolonize their teaching practices by drawing on their own histories of adaptation and resilience. They understand that their ability to move between two languages and cultures is not just an advantage but a critical element of creating inclusive learning spaces where students’ cultural and linguistic backgrounds are recognized as strengths rather than deficits. As Tejeda et al. (2003) argue, classrooms must serve as “sites for the development of critical consciousness” (p. 9), and the participants were well-positioned to foster this kind of awareness.

### 8.2. Decolonizing Teacher Education

The participants’ professional journeys also align with the broader call for decolonizing teacher education. Teacher education programs have the potential to serve as catalysts for moments of reflection and to advance a decolonial teaching practice that extends beyond mere discourse and suggests tangible actions that can be implemented in the English classrooms where these participants are working or plan to work. It entails experiencing pain or tension in the classroom as students learn to acknowledge their privilege in order to dismantle injustice. A key element of these decolonial practices is the utilization of lived experiences and transformative moments as “acts of healing.” This concept draws from Gloria Anzaldúa’s reflections in *This Bridge Called My Back* (Anzaldúa & Moraga, 1981), where she explores the interconnected paths that have brought us to the present, emphasizing the importance of forging connections and assuming responsibility for both personal and collective histories.

In this context, we call on educators to recognize and name the historical and contemporary wrongs committed against transnational youth, particularly those wrongs inflicted by Mexican educators who might otherwise be assumed to share an affinity with their Mexican-origin students. This acknowledgment is not about assigning blame but rather about creating spaces for dialogue about the unspoken injustices these future teachers may have experienced as transnational youth themselves (Bishop, 2019). In a classroom setting, small group discussions, followed by whole-class reflections, provide an avenue for students to process their own experiences and view healing as a form of survival and resilience. Author 1 and Author 2 have explicitly taught about transnationalism within English teacher education programs in Mexico, and we have witnessed firsthand the sense of relief expressed by transnational students when their experiences are validated, and when they are equipped with tools to support their future transnational students (Kasun & Mora-Pablo, 2021; Tacelosky, 2018). Given the increasingly transnational nature of the Mexican population (R. C. Smith, 2006), this conversation is not confined to the borders of the classroom but extends into everyday life.

Similarly, the participants’ reflections call into focus the need to have explicit instruction that allows students’ cultural and linguistic funds of knowledge to be centered and built upon in instruction. Esteban-Guitart et al. (2019) state that there must be concerted efforts in education to incorporate the “practices and life contexts of the learners,” going away from curriculums that “are often disconnected from the meaningful experiences, life contexts and practices of the learners.” These spaces challenge dominant practices and encourage the development of not often heard voices of students from minoritized communities. Translanguaging (García, 2009) allows students the use of both their native language and the target language as both languages are considered an integral part of the language process; they are interconnected (Herrera, 2023). Translanguaging recognizes the significance of students’ home language and is compatible with culturally relevant and sustaining pedagogy rooted in that they are “rooted children’s linguistic practices and cultural identities,” (Herrera, 2023).

There is room for much growth in both educational systems to embrace these approaches. No child should have to feel they cannot engage their linguistic and cultural backgrounds while attending school; on the contrary, they must be embraced for greater academic achievement.

### 8.3. Suggestions and Implications

A critical comparative analysis of education systems is essential for preparing future educators, particularly those who will work with migrant youth across borders. Educators often operate under systemic constraints, which can lead to misunderstandings and perceived negligence from students, such as Mexican-origin transnationals, who may feel unsupported by their teachers. However, it is crucial to contextualize these perceptions within the realities of educators’ working conditions. Teachers in Mexico, for instance, often face overwhelming workloads and economic limitations that prevent them from engaging in reflective, student-centered practices (Freire, 2000). This lack of time and resources exacerbates the challenges these educators face, leaving them ill-prepared to address the unique needs of transnational youth (Despagne & Jacobo Suárez, 2019).

The need for enhanced teacher education programs is evident. Current programs often fail to provide adequate training for working with transnational students, whose experiences and challenges require specialized understanding and pedagogical approaches (Frausto-Hernández, 2019). Without such training, teachers may struggle to provide equitable learning opportunities for these students, further perpetuating educational disparities. In the classroom, fostering an environment where students—particularly transnational youth—can share their experiences is a crucial step in deconstructing harmful narratives. Encouraging dialog and reflection allows students to critically engage with both historical and current realities, fostering solidarity and mutual understanding. As Anzaldúa (2015) reminds us, recognizing our interconnectedness—across lines of race, gender, and nationality—creates a foundation for more equitable and inclusive education. This kind of reflective, dialogic learning not only benefits transnational students but also prepares all students to think critically about their place within a globalized society. We explore the challenging yet hopeful field of educational teaching exchange across national borders between Mexico and the United States. The concept of ‘cross-pollination’ in teaching methods involves teachers from both borders influencing one another to improve their educational strategies by actively and contextually interacting rather than imitating each other. The initial step requires us to recognize the power dynamics and unequal structures present in cross-border comparisons. Kasun and Mora-Pablo (2022) observe that educators and institutions commonly accept the belief that U.S.-based teachers hold superior professional status. The belief about teaching quality stems from resource availability and institutional status rather than teaching methods thereby hiding important issues of fair education practices and cultural awareness. Mexican educators sometimes develop a mistaken version of decoloniality that replaces local knowledge systems with global (primarily Anglo-American) frameworks without evaluating their contextual applicability.

We also recognize the senses of market pressures (Kasun & Mora-Pablo, 2022) leading to future teachers’ choices to become educators as well as their senses of pride in having something that is worth actual money–their English skills. While bilingual education research has tended to acknowledge the foundational importance of language as a right and a marker of identity, our findings indicate it is at times a marker of potential earnings in an increasingly globalized world. We believe there are implications here regarding how educators decide to become educators and how that later impacts the ways they teach, in ways that could be advantageous and also perhaps more income than student focused. We cast no judgment on this possibility but argue it merits further study. What happens when the rhetoric about “love for language” is challenged with market-ready, language as lingua franca skill? This study offers comprehensive narrative insights into the construction of pedagogical identities and the envisioning of inclusive practices by transnational instructors; nevertheless, it lacks direct observation or analysis of their actual classroom practices as performed in other studies (see Frausto-Hernández, 2019). Consequently, our results are interpretive, reflecting the participants’ self-perceptions rather than being substantiated by actual teaching practices. This signifies a limitation of the present study and a significant direction for future investigation, as discussed in the following section.

## 9. Conclusions

The call for a decolonial approach to teacher education underscores the need for systemic reform in how future educators are prepared, particularly those who will teach in increasingly transnational contexts. Addressing the gaps in teacher training—particularly in relation to the needs of migrant and transnational youth—requires intentional collaboration between teacher education programs and institutions across borders. Concretely, we recommend that teacher education and teaching be better supported with better funding, in terms of resources for teacher education programs and for teachers themselves in terms of pay. This allocation of resources signals the seriousness of the field, and in that effort, we argue teachers need deeper preparation in terms of their understandings of transnational youth and the ways they can be better instructed. Specifically, for teacher preparation surrounding transnationalism, coursework related to culturally sustaining pedagogy (e.g., Paris & Alim, 2017) must also include discussions and application of knowledge related to transnational identities and the factors that create these push-and-pull phenomena related to migration. As a result, transnational identities can be highlighted by their teachers as valid, meaningful, and even sources of learning for the entire class. By comparing and implementing diverse decolonial practices, teacher preparation programs can develop culturally responsive pedagogies that transcend national boundaries.

We argue our findings have far-reaching implications. These tensions create extensive consequences that affect multiple areas. These tensions shape educators’ professional self-concept as well as their perspective on teaching as a transformative practice and their decision-making between income and educational impact. This statement does not imply a dichotomous choice. Educators face the challenge of maintaining their dedication to inclusive critical teaching that recognizes students’ realities while managing structural conditions in both regions. Cross-pollination needs to follow a dialogical approach rather than a hierarchical structure. The educational process needs to establish reciprocal learning which builds upon a mutual dedication to justice and multilingualism through the co-construction of knowledge.

Future research should focus on collaboration between teacher training programs in different regions of Mexico and other countries with high rates of transnational youth. Such collaborations will allow for the sharing of best practices and the development of a new, international paradigm for teacher education. This paradigm must equip educators with the tools to navigate the complexities of teaching in transnational contexts and address the unique needs of their students. In doing so, teacher education programs can contribute to the creation of more equitable educational systems worldwide, ensuring that all students—regardless of their migration status—have access to meaningful, culturally responsive education.

## Data Availability

The original contributions presented in this study are included in the article. Further inquiries can be directed to the corresponding author.
